# Training Population Optimization for Genomic Selection in *Miscanthus*

**DOI:** 10.1534/g3.120.401402

**Published:** 2020-05-26

**Authors:** Marcus O. Olatoye, Lindsay V. Clark, Nicholas R. Labonte, Hongxu Dong, Maria S. Dwiyanti, Kossonou G. Anzoua, Joe E. Brummer, Bimal K. Ghimire, Elena Dzyubenko, Nikolay Dzyubenko, Larisa Bagmet, Andrey Sabitov, Pavel Chebukin, Katarzyna Głowacka, Kweon Heo, Xiaoli Jin, Hironori Nagano, Junhua Peng, Chang Y. Yu, Ji H. Yoo, Hua Zhao, Stephen P. Long, Toshihiko Yamada, Erik J. Sacks, Alexander E. Lipka

**Affiliations:** * Dept. of Crop Sciences, University of Illinois, Urbana, IL; † Plant Genome Mapping Laboratory, University of Georgia, 111 Riverbend Road, Athens, GA 30605; ‡ Applied Plant Genome Laboratory, Research Faculty of Agriculture, Hokkaido University, Japan; § Field Science Center for Northern Biosphere, Hokkaido University, Sapporo, Hokkaido 060-0810, Japan; ** Department of Soil and Crop Sciences, Colorado State University, Fort Collins, CO 80523; †† Department of Applied Bioscience, Konkuk University, Seoul 05029, South Korea; ‡‡ Department of Biochemistry, University of Nebraska-Lincoln, NE 68588; §§ Department of Applied Plant Science, Kangwon National University, Chuncheon 24341, South Korea; *** Department of Agronomy, Zhejiang University, Hangzhou 310058, China; ††† China National Seed Group Co. Ltd, Wuhan, Hubei 430040, China; ‡‡‡ Department of Applied Plant Sciences, Kangwon National University, Chuncheon, Gangwon 200-701, South Korea; §§§ College of Plant Science and Technology, Huazhong Agricultural University, Wuhan, Hubei 430070, China; **** Vavilov All-Russian Institute of Plant Genetic Resources, 42–44 Bolshaya Morskaya Street, 190000 St. Petersburg, Russia

**Keywords:** Miscanthus, Prediction Accuracy, Genomic selection, Population Structure, GenPred, Shared data resources

## Abstract

*Miscanthus* is a perennial grass with potential for lignocellulosic ethanol production. To ensure its utility for this purpose, breeding efforts should focus on increasing genetic diversity of the nothospecies *Miscanthus* × *giganteus* (M×g) beyond the single clone used in many programs. Germplasm from the corresponding parental species *M. sinensis* (Msi) and *M. sacchariflorus* (Msa) could theoretically be used as training sets for genomic prediction of M×g clones with optimal genomic estimated breeding values for biofuel traits. To this end, we first showed that subpopulation structure makes a substantial contribution to the genomic selection (GS) prediction accuracies within a 538-member diversity panel of predominately Msi individuals and a 598-member diversity panels of Msa individuals. We then assessed the ability of these two diversity panels to train GS models that predict breeding values in an interspecific diploid 216-member M×g F_2_ panel. Low and negative prediction accuracies were observed when various subsets of the two diversity panels were used to train these GS models. To overcome the drawback of having only one interspecific M×g F_2_ panel available, we also evaluated prediction accuracies for traits simulated in 50 simulated interspecific M×g F_2_ panels derived from different sets of Msi and diploid Msa parents. The results revealed that genetic architectures with common causal mutations across Msi and Msa yielded the highest prediction accuracies. Ultimately, these results suggest that the ideal training set should contain the same causal mutations segregating within interspecific M×g populations, and thus efforts should be undertaken to ensure that individuals in the training and validation sets are as closely related as possible.

Current global reliance on fossil fuels is one of the main drivers of climate change (Dale *et al.* 2011), the physical impact of which is already being observed. It is therefore imperative that alternate sources of energy be utilized to meet current energy demands. One such source is bioenergy crops, which offer more sustainable energy through reduced carbon footprints (Saha *et al.* 2013). Among the biofuel crops currently being evaluated, *Miscanthus*, a C_4_ perennial grass that is a phylogenetically close relative to sugarcane (*Saccharum officinarum*), has great potential for combustion and lignocellulosic ethanol biofuel production, as well as abundant genetic diversity for climatic adaptation (Clifton-Brown *et al.* 2008a; Dwiyanti *et al.* 2013; Sacks *et al.* 2013). To date, most breeding efforts for the development of *Miscanthus* as a bioenergy crop have focused on the nothospecies *M*. × *giganteus* (M×g), an interspecific cross between *M. sinensis* (Msi, which is predominantly diploid) and *M. sacchariflorus* (Msa, which has diploid and tetraploid forms) (Hodkinson *et al.* 2002).

Virtually all M×g accessions used for bioenergy in North America and Europe are derived from the same infertile clone, ‘1993-1780’ (Clifton-Brown *et al.* 2008; Glowacka *et al.* 2014; Heaton *et al.* 2008). Reliance on this clone has significant drawbacks for further development because it displays insufficient winterhardiness in northern latitudes of North America (Dong *et al.* 2019) and flowers too early in southern latitudes of the US (Erik Sacks, unpublished data), thereby having substantial negative impact on yield. Thus, there is a critical need to increase genetic diversity of mainstream M×g biofuel crops. Breeding efforts are currently underway to incorporate the copious genetic diversity of Msi and Msa into new M×g clones (Clifton-Brown *et al.* 2019). In particular, it should be possible to use genome-wide marker data available in both Msi and Msa in genomic selection (GS) models to predict which M×g clones have optimal breeding values. By leveraging genomic and phenotypic data in a training set, GS is capable of predicting genomic-estimated breeding values (GEBVs) of unphenotyped related accessions based solely on genome-wide marker data (Meuwissen *et al.* 2001; Jarquín *et al.* 2017). Given that previous GS studies in Msi obtained moderate- to high prediction accuracies for biomass and cell wall composition (Slavov *et al.* 2014; Davey *et al.* 2017; Clark *et al.* 2019b), GS is theoretically well-suited to utilize the genetic diversity of existing Msi and Msa diversity panels and select genetically distinct M×g clones with superior biofuel capacity.

The task of training a GS model on Msi and Msa diversity panels and then using it to predict phenotypically optimal M×g clones presents some challenges that need to be explored. The first is that subpopulation structure is prominent in both Msi and Msa (Clark *et al.* 2014, 2018), which could result in a bias in the prediction accuracies from GS models trained on these data (Guo *et al.* 2014). Such unaccounted population structure could also have a major impact on the efficiency of incorporating GS into breeding programs (Windhausen *et al.* 2012; Isidro *et al.* 2015). One approach for mitigating these adverse impacts of population structure on GS breeding programs is to identify an optimal subset of individuals with minimal relatedness for training a GS model. In particular, the coefficient of determination mean (CDmean) criterion (Rincent *et al.* 2012, 2017) tends to select individuals that span the range of genetic diversity present in a given diversity panel, thereby resulting in a training set of individuals with a minimal amount of relatedness to one another.

The second challenge to consider is the impact of using diversity panels from two related species (Msi and Msa) to train a GS model for predicting breeding values in interspecific M×g breeding material. Previous work has shown that the degree of relatedness between individuals in the training set and the breeding material has a major influence on GS prediction accuracies, where highest accuracies tend to be achieved when the individuals in both sets are highly related (Clark *et al.* 2012; Windhausen *et al.* 2012; Wientjes *et al.* 2013; Lorenz and Smith 2015). Thus, the impact of using a training set consisting of diversity panels from two different species on GS prediction accuracy needs to be carefully explored and quantified. In particular, a systematic comparison of using both Msi and Msa diversity panels to train a GS model *vs.* using each panel separately will help determine the extent to which the additional degree of genetic diversity afforded by using both panels impact the accurate prediction of breeding values in M×g clones.

In summary, the development of optimal training sets will enable a propitious utilization of the genetic diversity in Msi and Msa for predicting GEBVs of traits related to biofuel capacity and winter hardiness in M×g breeding populations. This could enhance current efforts to incorporate more genetic diversity into M×g clones by applying GS as effectively and efficiently as possible. Therefore, the purpose of this study was to explore the influence of Msi and Msa training set composition on the ability of GS models to predict breeding values of M×g clones. We considered a prediction set consisting of a diploid F_2_ population derived from an Msa × Msi cross, as well as 50 simulated interspecific diploid F_2_ populations derived from different sets of diploid parents that were randomly selected from Msi and Msa diversity panels. The latter simulation study was conducted to mitigate the drawback of having only one interspecific diploid F_2_ population available for analysis. Our specific objectives were to (i) evaluate the impact of population structure in Msi and Msa diversity panels on GS prediction accuracy and (ii) quantify the advantages and disadvantages of using both Msi and Msa panels as a training set for fitting GS models.

## Materials and Methods

We used both observed and simulated genotypic and trait data to investigate the ability of an Msi diversity panel (Clark *et al.* 2019a) and an Msa diversity panel (Clark *et al.* 2018) to accurately predict breeding values in an interspecific diploid F_2_ population (Kaiser and Sacks 2015). The observed traits were used to evaluate the impact of population structure on GS prediction accuracies within each diversity panel, as well as to assess the ability of various subsets of the diversity panels to predict interspecific F_2_ breeding values. To quantify the stability of prediction accuracies in the F_2_ population, a complementary simulation study was conducted. Specifically, 50 interspecific diploid F_2_ populations were simulated (where each population was derived from a different set of randomly selected parents), and then simulated traits were used to assess the ability of the Msi and Msa panels to train GS models for accurate prediction of GEBVs in all 51 F_2_ populations.

### Field trials and phenotypic analysis

Phenotypic data, including basal circumference (Bcirc), compressed circumference (Ccirc), culm length (CmL), diameter of basal internode (DBI), number of days to first heading (HD1), and dry biomass yield (Yld), were obtained during the third year of three panels grown at replicated field trials. Each panel was planted as a randomized complete block design at multiple locations, with genotypes propagated vegetatively as clonal replicates. Due to import restrictions and differential survival of genotypes, not all genotypes were able to be evaluated at all locations.

The first panel evaluated was a diversity panel of Msi, previously described by Clark *et al.* (2019a). These evaluations took place at five temperate locations, including Sapporo, Hokkaido, Japan (HU), Leamington, Ontario, Canada (NEF), Fort Collins, Colorado, USA (CSU), Urbana, Illinois, USA (UI), and Chuncheon, South Korea (KNU), with four blocks per location. All locations included a trial planted in 2012 and evaluated in 2014, with an additional trial at KNU planted in 2013 and evaluated in 2015. Data from a subtropical location (Zhuji, China) were omitted from this study. In total 530 diploid genotypes of Msi were evaluated, as well as six diploid and two tetraploid genotypes of Msa.

The second panel evaluated a diversity panel of Msa. These trials took place at three temperate locations, including HU (four blocks), UI (four blocks), and KNU (one block). Trials were planted in 2015 and evaluated in 2017. In total 356 diploid and 242 tetraploid genotypes of Msa were evaluated, including the six diploid and two tetraploid genotypes that were also evaluated in the Msi trials. Population structure and genetic diversity of the genotypes within the diversity panel have been described by Clark *et al.* (2018).

Lastly, the third panel was a diploid F_2_ population derived from Msa ‘Robustus’ × Msi ‘Cosmopolitan Revert’ (called the “09F2” population). This population was evaluated in a single field trial with four blocks planted at UI in 2015 and evaluated in 2017. This population has been previously described by Kaiser and Sacks (2015) and Dong *et al.* (2017). ‘Cosmopolitan Revert’ was also evaluated in the Msi diversity panel trial, and ‘Robustus’ in both the Msi and Msa diversity panels. Trait data were obtained for 216 F_2_ progeny.

Across all three panels and locations, least-squared means (LSmeans) of the six evaluated traits for each clonal genotype were estimated using the emmeans R package (Lenth *et al.* 2019) using the following model:

$$Y_{ijk}=\mu +G_{i}+T_{j}+{\left(GT\right)}_{ij}+B{\left(T\right)}_{k\left(j\right)}+\varepsilon_{ijk},$$

where $Y_{ijk}$ is the Box-Cox transformed trait (Box and Cox 1964) of the plot in the $k^{th}$ block in the $j^{th}$ trial (*i.e.*, each combination of location, year, and panel) with the $i^{th}$ clonal genotype, μ us the grand mean, $G_{i}$ is the fixed main effect of the $i^{th}$ clonal genotype, $T_{j}$ is the random main effect of the $j^{th}$ trial, ${\left(GT\right)}_{ij}$ is the random two-way interaction effect between the $i^{th}$ clonal genotype and the $j^{th}$ trial, $B{\left(T\right)}_{k\left(j\right)}$ is the random effect of the $k^{th}$ block nested within the $j^{th}$ trial, and $\varepsilon_{ijk}$ is the random error term associated with the plot in the $k^{th}$ block in the $j^{th}$ trial with the $i^{th}$ clonal genotype. For the Box-Cox transformation, the optimal lambda values selected were 0.4 for Ccirc and Bcirc, 1.2 for CmL, 0.2 for DBI, and 0.3 for Yld. The trait HD1 was left untransformed.

### Genetic marker data

All *Miscanthus* genotypes were subjected to RAD-seq, using the protocol described by Clark *et al.* (2014). The DNA from the individuals in the Msi and Msa diversity panels were digested with *Pst*I and *Msp*I and sequenced adjacent to the *Pst*I site, whereas *Sbf*I was used in place of *Pst*I for DNA from the individuals in the 09F2 population. In all cases, a 200-500 bp size selection was used to reduce library complexity.

A custom pipeline using TASSEL5 GBSv2 (Glaubitz *et al.* 2014), Python, and R was used to find common markers among the three trials and call genotypes. The pipeline is available at https://github.com/lvclark/TASSELGBS_combine (DOI: 10.5281/zenodo.3367470). Briefly, in each population, TASSEL5 GBSv2 was used to identify unique tags and their read depth within each taxon. Bowtie2 (Langmead and Salzberg 2012) was then used to align the tags to the *Miscanthus sinensis* v7.1 reference available from DOE-JGI at https://phytozome.jgi.doe.gov/. A Python script using TagDigger (Clark and Sacks 2016) was then used to import SAM files, find common alignment locations among the three datasets, organize tags into markers, and filter markers based on missing data rate and minor allele frequency (MAF). For the Msi and Msa diversity panels, each marker had to have at least 300 individuals with reads and five individuals with the minor allele. For the 09F2 population, each marker had to have at least 200 individuals with reads and at least 30 individuals with the minor allele. An R script using polyRAD (Clark *et al.* 2019c) was then used for genotype calling, and a matrix of posterior mean genotypes, scaled from 0 to 1, was exported for the combined dataset. The subsequent set of 5,140 markers were used to fit and evaluate all GS models considered in the analysis of the observed traits. Using these markers, narrow-sense heritabilities were estimated for each evaluated trait within each of the three panels by dividing genetic variance component estimate by the sum of genetic variance and residual variance component estimates from the *mixed.solve* function in the rrBLUP R package (Endelman 2011).

A second filtering procedure was conducted to obtain markers for the simulation study. This procedure used the same markers segregating in the three panels, but the genotypes called by polyRAD were discretized to allow for the quantification of dominance effects, and hence assess the importance of dominance in the downstream GS analysis. Another round of filtering was conducted to remove markers that were not anchored to a genetic map created for the 09F2 population (Dong *et al.* 2018) (https://onlinelibrary.wiley.com/doi/full/10.1111/gcbb.12472); such a filtering step was essential because a genetic map was needed to create the 50 simulated interspecific F_2_ populations. Upon completion, a total of 356 markers across 19 linkage groups were available for the simulation study.

### Quantifying subpopulation structure within diversity panels

To quantify subpopulation structure, a principal component analysis (PCA) of the genomic markers (Price *et al.* 2006) was conducted separately within the Msi and Msa diversity panels using the *prcomp()* R funciton. This PCA was conducted in both panels using the same 5,140 markers used to evaluate the performance of GS on the observed phenotypic data to facilitate an assessment of the contribution of population structure to GS prediction accuracies. Kinship matrices for accessions within the Msi and Msa diversity panels and 09F2 breeding population was generated using the popkin R package (Ochoa and Storey 2019).

### GS model and quantification of prediction accuracy

The GS model employed to analyze the observed and simulated trait data were the random regression best linear unbiased prediction (RR-BLUP) model (Whittaker *et al.* 2000; Meuwissen *et al.* 2001), and has been previously described (*e.g.*, in Rice and Lipka 2019). In brief, this model is written as follows:

$$Y_{i}=\beta_{0}+{\text{Σ}}_{j=1}^{p}\beta_{j}x_{ij}+\varepsilon_{ij},$$

where $Y_{i}$ represents the LSMean of the observed trait or the simulated trait from the $i^{th}$ individual, $\beta_{0}$ is the intercept parameter, $\beta_{j}$ is the random additive effect of the $j^{th}$ marker ∼N(0,$\sigma_{\beta }^{2}$), $x_{ij}$ is the observed marker genotype of the $j^{th}$ marker at the $i^{th}$ individual (enumerated as -1 and 1 for the homozygous genotypes and 0 for the heterozygous genotype), and $\varepsilon_{i}$ is the random error term for the $i^{th}$ individual ∼N(0,$\sigma_{E}^{2}$). The BLUP of $\beta_{j}$ is subjected to the ridge regression penalty (Hoerl and Kennard 1970). This model was fitted using the rrBLUP R package (Endelman 2011). For a given training and validation set considered in this study, prediction accuracy was assessed by fitting a GS model in the training set and then estimating the Pearson correlation coefficient between the observed trait LSMeans or simulated trait values and the GEBVs among the individuals in the validation set.

### Assessing the impact of population structure within diversity panels on GS prediction accuracy

**Figure 1:**
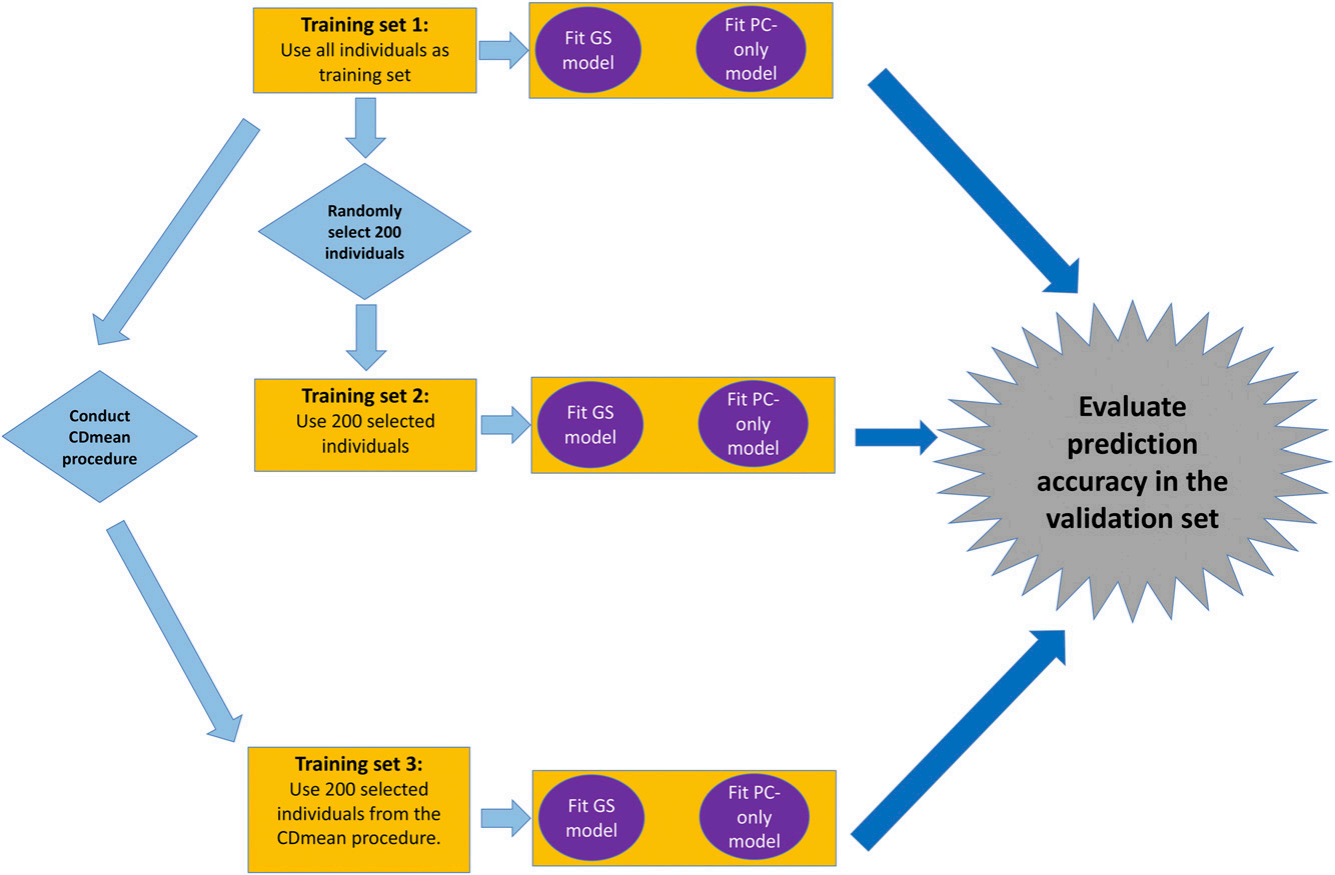
Schematic representation of the methods to account for population structure within *Miscanthus sinensis* and *Miscanthus sacchariflorus* diversity panels. Yellow rectangles refer to a given training set of individuals obtained from a fivefold cross validation procedure conducted within each diversity panel. Purple circles refer to the models that are trained; specifically the genomic selection (GS) model is a random regression best linear unbiased prediction (RR-BLUP) model, and the principal components (PC)-only model includes only the top PCs of 5,140 genome-wide markers as explanatory variables (see Figure S1 for scree plots). The blue diamonds refer to the process of randomly selecting 200 individuals and conducting CDmean procedure. Prediction accuracy, quantified as the Pearson correlation between the observed phenotypic values and the genomic estimated breeding values (GEBVs) from each GS model, is then calculated among individuals in the corresponding validation set (gray symbol).

We evaluated two strategies to quantify the influence of population structure on the performance of GS on the observed traits within the Msi and Msa diversity panels, which are summarized in Figure 1. To assess the influence of population structure on GS prediction accuracy, we compared the prediction accuracy of the RR-BLUP model for each trait to a corresponding model that included the LSMean of the trait as response variable and the top PCs explaining the greatest amount of marker variance as the explanatory variables (the specific number of PCs were seven for Msi and Msa, shown in Figure S1 A-B). To quantify the impact of the training set composition on GS prediction accuracies, we performed ten replicates of a fivefold cross validation procedure (Kohavi 1995) within each diversity panel. The individuals of each training set obtained from the fivefold cross validation procedure were used to assess the impact of training set composition on GS prediction accuracies. That is, either (i) all individuals in each training set was used to fit each GS model, (ii) 200 randomly selected individuals in each training set were used to fit each GS model, or (iii) all individuals in each training set served as a calibration set for using the CDmean procedure (Laloë 1993; Rincent *et al.* 2012) to obtain 200 optimized individuals for fitting each GS model. The purpose of sets (ii) and (iii) was to compare the prediction accuracy of a set chosen by the CDmean procedure to a random set of individuals of the same sample size. Thus, for each training set from the fivefold cross-validation procedure, GS models were fitted to the subsets of individuals described in (i), (ii), and (iii), and then prediction accuracy was assessed in the corresponding validation set.

The CDmean procedure has been previously described (Rincent *et al.* 2012, 2017). In brief, this procedure identifies sets of individuals that maximizes the mean squared correlation of the actual and predicted (from the GEBVs) differences between each unphenotyped individuals’ trait value and the population mean trait value. Each time the CDmean procedure was conducted, a random subset of 200 individuals in the training set was initially selected, and then the “Algo1” exchange algorithm described in Rincent *et al.* (2012) was conducted 3,000 times to identify a set of 200 individuals where the CDmean value was maximized.

### Assessing the impact using both Msi and Msa as a training set on GS prediction accuracy

We next assessed the ability of the Msi and Msa panels to obtain accurate GEBVs of the observed trait LSMeans in the 09F2 population. Seven different configurations (summarized in Table 1) of individuals from the Msi and Msa diversity panels were used as training sets. The GEBVs from the Whole.MsiMsa scenario listed in Table 1 were calculated as the sum of GEBVs from a model trained in the entire Msi panel and GEBVs from a model trained in the entire Msa panel. For each of these configurations, prediction accuracy was assessed on 1,000 bootstrap samples of 216 individuals from the 09F2 population.

**Table 1:** Training populations derived from the Miscanthus sinensis (Msi) and Miscanthus sacchariflorus (Msa) diversity panels that were used to train genomic selection models fitted to predict trait values in the 09F2 population. Note that the CDmean procedure will select different subsets of individuals for each trait.

Scenario	Individuals from Msi panel used in training set	Individuals from Msa panel used in training set
Msi.Random	200 Randomly selected individuals from Msi panel	None
Msa.Random	None	200 Randomly selected individuals from Msa panel
Msi.CDmean	200 Msi individuals selected from CDmean procedure	None
Msa.CDmean	None	200 Msa individuals selected from CDmean procedure
Msi.Whole	Entire Msi panel	None
Msa.Whole	None	Entire Msa panel
Whole.Msi.Msa	Entire Msi panel	Entire Msa panel

### Assessment of robustness across multiple interspecific F_2_ families via simulation study

Given that the 09F2 population was the only interspecific F_2_ population available for making inferences, we simulated 50 interspecific F_2_ populations to further evaluate the ability of diversity panels to predict GEBVs in breeding populations. Each interspecific F_2_ population consisted of one randomly selected Msi parent, and one randomly selected diploid Msa parent. The simulation of each F_2_ population began with the random selection of one Msi and one diploid Msa parent from the respective diversity panels. For each set of parents, 50 F_1_ individuals were simulated and then intermated to simulate 216 F_2_ individuals. The protocol implemented to simulate these individuals has been previously described (Olatoye *et al.* 2019). In brief, normal meiotic segregation was assumed and a custom Haldane mapping function was used to simulate crossovers based on the aforementioned genetic map of 356 markers in the 09F2 population. Upon completion, a total of 50 interspecific F_2_ families, each consisting of 216 individuals, were available for evaluation.

A custom script in the R programming language similar to the one described in Olatoye *et al.* (2019) was used to simulate traits in each diversity panel and F_2_ population. This script randomly selected a subset of the 356 discrete markers to be quantitative trait nucleotides (QTNs) underlying these traits. The genetic architectures (*i.e.*, configurations of number of QTNs, as well as how many of them were additive, dominant, or additive x additive epistatic) of the simulated traits varied according to five different scenarios presented in Table 2. Three of these scenarios (Scenarios 1,2, and 3 presented in Table 2) considered QTNs with effect sizes that were equal across both panels were simulated. For these scenarios the same number of QTNs were randomly selected and assigned the same effect size, the latter of which was determined using the *runif()* function in R. Two scenarios (Scenarios 4 and 5) considered the impact of unequal QTN effect sizes between the two different species on prediction accuracies. Finally, the impact of different QTNs segregating within the diversity panels were assessed by selecting QTNs based on the following: (i) the same set of QTNs were selected for both diversity panels (Scenario 1), (ii), different QTNs were selected within each diversity panel (Scenario 3, 4, and 5), and (iii) QTNs were selected from each panel such that 50% of them were the same while 50% were different (Scenario 2). For each F_2_ population, all segregating QTNs from both diversity panels contributed to the genetic signal of the simulated trait.

**Table 2:** Description of trait simulation in the Miscanthus sinensis (Msi) and Miscanthus sacchariflorus (Msa) diversity panels and simulated 50 F2 populations that were used to perform genomic selection

Scenario	Description	QTN*^a^* Effect	Model + QTN #*^b^*	*H^2^* Panel*^c^*	*H^2^* F_2_
1	The same QTNs in Msi and Msa	Random uniform between 0 and 1 (Same effects in both Msi and Msa)	A20D0E0, A20D4E0, A20D0E4	0.60	0.37
2	Half of the QTNs similar and half different between Msi and Msa	Random uniform between 0 and 1 (Same effects in both Msi and Msa)	A20D0E0, A20D4E0, A20D0E4	0.60	0.37
3	Completely different QTNs used in Msi and Msa	Random uniform between 0 and 1 (Same effects in both Msi and Msa)	A20D0E0, A20D4E0, A20D0E4	0.60	0.37
4	Different QTNs with Large Effects in Msi	QTN from Msi with large effects (random uniform between 0.5 and 0.99) and Msa with small effects (random uniform between 0 and 0.25)	A20D0E0, A20D4E0, A20D0E4	0.60	0.37
5	Different QTNs with Large Effects in Msa	QTN from Msa with large effects (random uniform between 0.5 and 0.99) and Msi with small effects (random uniform between 0 and 0.25)	A20D0E0, A20D4E0, A20D0E4	0.60	0.37

a QTN, Quantitative trait nucleotide.

b A, D, and E respectively refer to additive, dominance, and additive-by-additive epistatic QTNs. The number after each letter refers to the number of QTNs with that genetic mechanism simulated. For example A20D0E0 means that 20 additive QTN were simulated, 0 dominance QTN were simulated, and 0 epistatic QTN were simulated.

c *H^2^*, broad-sense heritability.

The approaches used to assess the prediction accuracy across the 51 F_2_ populations were similar to those described for evaluating GS performance in the 09F2 observed trait data. In particular, the RR-BLUP GS models were trained using the same seven configurations of Msi and Msa diversity panel individuals described in Table 1. For each simulated trait and configuration, the observed prediction accuracy in the 09F2 population was compared to the distribution of prediction accuracies from the 50 simulated F_2_ populations.

### Data availability

R code, sequence data, phenotypic data, and individuals selected by CDmean during analysis are available at https://github.com/marcbios/MsiMsa. Supplemental material available at figshare: https://doi.org/10.25387/g3.12357563.

## Results

### Proportion of heritable variation was higher in diversity panels than in breeding population

We compared the distribution and heritabilities of the LSmeans of six observed traits measured in the Msi, Msa, and 09F2 panels. With the exception of Bcirc, the range of observed values of a given trait tended to be similar across the three panels (Figure S2 and Table S1). The distributions of HD1 was distinctively trimodal in the Msa panel, while all remaining distributions were generally unimodal. Finally, all six traits were less heritable in the 09F2 population than in the two diversity panels (Figure S3). This could suggest that only a subset of the genes comprising the genetic architecture of these evaluated traits were segregating in the 09F2 population.

### Principal component analyses confirmed presence of subpopulation structure in Msi and Msa diversity panels

The results of the PCA conducted on the genome-wide markers within each diversity panel underscore previous findings (Clark *et al.* 2014, 2018) that subpopulation structure is ubiquitous in both Msi and Msa. This result is illustrated in Figure S4, where the first two PCs within each panel clearly subdivided the accessions into distinct clusters that were consistent with the geographical origin of each accession. In addition, the kinship matrices showed that individuals in Msi and Msa panels formed two distinct groups and both had partial relationship with 09F2 based on the kinship values (Figure S5). Thus, these results suggest that subpopulation structure is one of the main drivers of marker variability in these two panels, and our exploration into the influence of this source of variability into GS prediction accuracy is justified.

### Subpopulation structure had a substantial impact on prediction accuracies within diversity panels

**Figure 2:**
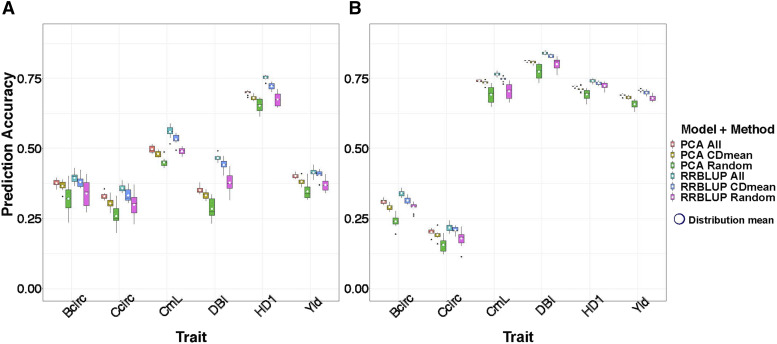
Prediction accuracy for within diversity panel genomic selection. Each boxplot represents the distribution of prediction accuracy (Y-axis) across ten replicates of fivefold cross-validation folds for each method (color coded) and for each trait (X-axis), specifically basal circumference (Bcirc; cm), compressed circumference (Ccric; cm), culm length (CmL; cm), diameter of basal internode (DBI; mm), days to first heading (HD1; days), and yield (Yld; g/plant) for (A) *Miscanthus sinensis*, (B) *Miscanthus sacchariflorus*. RR-BLUP refers to the random regression best linear unbiased prediction model, while PCA refers to the model where the trait is the response variable and the top principal components of a principal component analysis of 5,140 markers are used as explanatory variables. CDmean refers to the subset of 200 individuals selected using the CD mean procedure, while Random refers to a random subset of 200 individuals. The white dots represent the mean value of each distribution.

For each of the observed traits in each diversity panel, the prediction accuracies of the PCA model were similar, albeit less than those from the corresponding RR-BLUP model (Figure 2). This suggests that a non-negligible proportion of the genomic variability captured in these RR-BLUP models were from subpopulation structure within each diversity panel. The differences in prediction accuracy between the PCA and RR-BLUP models were more variable across traits in the Msi panel than in the Msa panel, which could indicate that the influence of population structure on RR-BLUP prediction accuracies for these observed traits was more constant in the Msa panel.

The prediction accuracies from the models fitted using 200 individuals selected from the CDMean procedure and models fitted using the random samples of 200 individuals were consistently less than models fitted from all individuals in the training set. However, the prediction accuracies from the model trained with 200 individuals selected from the CDMean procedure were uniformly higher than those trained from the random sample of 200 individuals. This suggests that individuals selected from the CDMean procedure could be better at predicting total genetic merit than an equally-sized random set of individuals, although the best prediction accuracies can be expected when all individuals in the training set are used.

### Training genomic selection models with diversity panels resulted in poor prediction of breeding population

**Figure 3:**
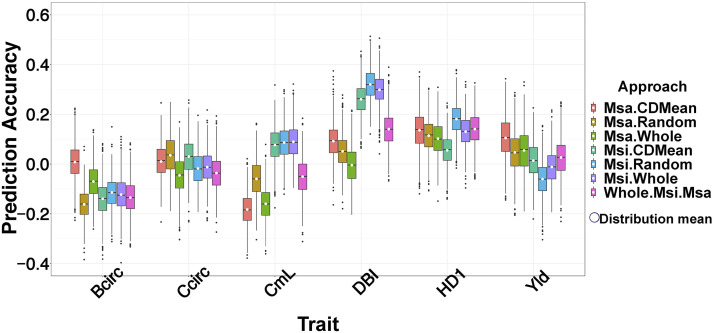
Prediction accuracy for using *Miscanthus sinensis* (Msi) and *Miscanthus sacchariflorus* (Msa) diversity panels to train genomic selection (GS) models for prediction in 09F2 breeding population. Each boxplot represents the distribution of prediction accuracy (Y-axis; across 1,000 bootstraps of the 09F2 breeding population) when the genomic selection model was trained using 200 individuals selected using CDMean (Msa.CDMean, Msi.CDMean), 200 randomly selected individuals (Msa.Random, Msi.Random), whole diversity panels (Msa.Whole, Msi.Whole) and a combination of the GEBVs estimated from Msi and Msa panels (Whole.Msi.Msa). The evaluated traits (X-axis) include basal circumference (Bcirc; cm), compressed circumference (Ccric; cm), culm length (CmL; cm), diameter of basal internode (DBI; mm), days to first heading (HD1; days), and yield (Yld; g/plant). The white dots represent the mean value of each distribution.

Regardless of which subset of individuals from the Msi and Msa diversity panels were used to train the GS model (summarized in Table 1), low prediction accuracies were obtained in the diploid 09F2 population (Figure 3). Interestingly, the configuration of adding the GEBVs from models trained separately in the Msi and Msa panels did not yield the highest prediction accuracies. Depending on the trait, models trained in either the Msi or Msa panels alone resulted in the highest prediction accuracies. With the exception of the Msa panel being used to train GS models for Bcirc, use of the CDMean procedure to select an optimal training set did not substantially increase prediction accuracies relative to using an entire diversity panel or a random sample of 200 individuals as a training set.

### Sum of GEBVs From models trained in Msi and Msa panels yielded highest prediction accuracies in simulated F_2_ populations

**Figure 4:**
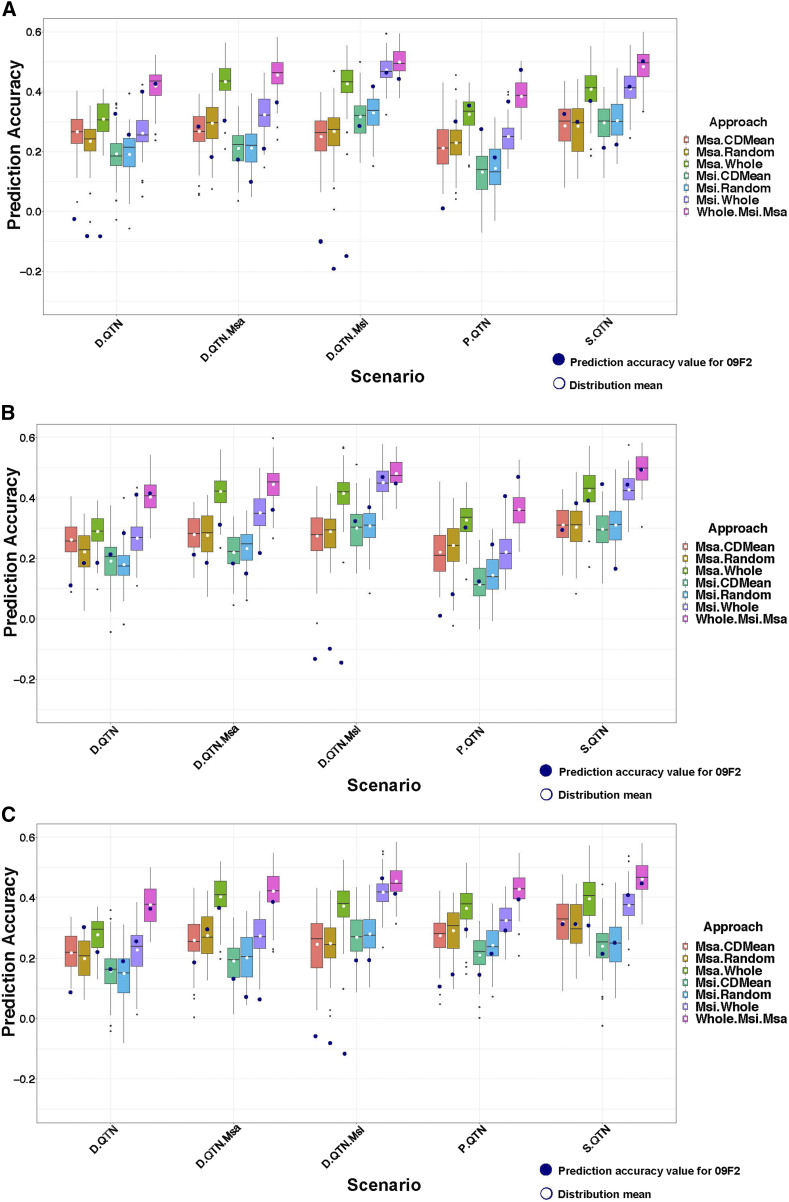
Prediction accuracy for using *Miscanthus sinensis* (Msi) and *Miscanthus sacchariflorus* (Msa) diversity panels to train GS models for making predictions in simulated F_2_ populations. Boxplots represent a distribution of prediction accuracies (Y-axis) across 50 simulated interspecific F_2_ populations for simulated traits (X-axis). Boxplots are color coded according to approaches used to select training sets: 200 individuals selected randomly (Msa.Random, Msi.Random) or using the CDMean procedure (Msa.CDMean, Msi.CDMean), whole diversity panels (Msa.Whole, Msi.Whole) and the sum of the genomic estimated breeding values (GEBVs) estimated from GS models fitted separately within the Msi and Msa panels (Whole.Msi.Msa). Traits were simulated using five different scenarios namely; D.QTN (traits simulated with completely different QTN in Msi and Msa but with the same effect sizes), D.QTN.Msa (traits simulated with different QTNs in each of Msi and Msa, with Msa QTNs having large effects while Msi QTNs had small effects), D.QTN.Msi (traits simulated with different QTNs in each of Msi and Msa, with Msi QTNs having large effects while Msa QTNs had small effects), P.QTN (traits where 50% of the QTNs were the same across Msi and Msa, while 50% were different), and S.QTN (traits simulated in Msi and Msa based on the same QTNs and same effect sizes). Three different combinations of additive and non-additive QTNs were considered, specifically (A) traits with 20 additive QTN, 0 dominance QTN, and 0 epistatic QTN, (B) traits with 20 additive QTN, 4 dominance QTN, and 0 epistatic QTN, and (C) traits with 20 additive QTN, 0 dominance QTN, and 4 epistatic QTN. The white dots represent the mean value of each distribution while the black dot represent the prediction accuracy value for the same simulated genetic architecture using polyRAD genetic data in the 09F2 population.

Similar distributions of prediction accuracies were obtained from the 50 simulated F_2_ populations regardless of how many QTNs were the same across the Msi and Msa panels, or if non-additive QTNs were simulated (Figure 4). Across all genetic architectures that were considered, the sum of GEBVs from models trained separately within the entire Msi and Msa panels yielded the highest prediction accuracies. However, for the scenarios where only one of the panels contained large-effect QTNs (denoted “D.QTN.Msa” and “D.QTN.Msi” on the X-axes in Figure 4), GEBVs from panels containing these large-effect QTNs were nearly as accurate as the sum of GEBVs from both panels. Moreover, the GEBVs from GS models trained on 200 individuals selected from the CDMean procedure were never as accurate as models trained from all individuals from the same corresponding panel, nor were they discernably different from models trained from random subsets of 200 individuals. Finally, the sum of GEBVs from GS models trained in the Msi and Msa panels consistently produced prediction accuracies for the 09F2 population that were most similar to the distributions of prediction accuracies across the 50 simulated F_2_ populations.

## Discussion

The exploitation of Msi and Msa diversity for genomic prediction of M×g clones will not reach its full potential until the characteristics of an optimal training set for a target trait are identified. To this end, we evaluated the degree to which GS prediction accuracies of six biofuel-related traits within Msi and Msa diversity panels were influenced by subpopulation structure, and then we assessed the usefulness of including individuals from both of these panels to train GS models that predict breeding values in an interspecific diploid M×g F_2_ population. To overcome the pitfall of having only one interspecific F_2_ population available for analysis, we also conducted a simulation study to assess the empirical distributions of prediction accuracies from GS models trained in these diversity panels across 50 simulated interspecific diploid M×g F_2_ populations. Each of these simulated populations were derived from crossing different randomly selected Msi and diploid Msa parents. Our results demonstrated that (i) unaccounted population structure can predict breeding values nearly as well as an RR-BLUP model within each of the panels, (ii) there are differences in the predictive ability of the diversity panels and such differences are likely to be trait-dependent, and (iii) the combination of GEBVs independently estimated from Msi and Msa resulted in increased prediction accuracies for the simulated data, but not for the observed trait data.

### Impact of subpopulation structure on prediction accuracies within diversity panels

Our analysis of the observed traits suggests that subpopulation structure contributed substantially to the prediction accuracies within each panel (Figure 2), and this finding is consistent with previous studies conducted by ourselves and others (Clark *et al.* 2019b; Crossa *et al.* 2016; Wientjes *et al.* 2015). When we previously performed genomic selection on the Msi panel using a larger set of 46,177 genome-wide SNPs, we observed decreases in prediction accuracy after accounting for subpopulation structure (Clark *et al.* 2019b). Outside of Msi, Crossa *et al.* (2016) reported a decrease of 15–20% when using PCs of markers in the GS model to account for population structure present in two collections of wheat landrace accessions, while Wientjes *et al.* (2015) observed that using population information among three cattle breeds for prediction gave prediction accuracies up to 30% higher than those from a GS model including breed as a fixed effect. We interpret these collective findings as suggesting that population structure is an integral component of the genomic signal underlying these previously published traits, as well as the observed traits analyzed in our study. Thus, we recommend against factoring out population structure in practice because doing so could lead to the implementation of GS models that do not encapsulate the entire genomic contribution to these six biofuel traits.

Another area of future research is to explore the predictive ability of GS models that explicitly quantify different marker effects within each subpopulation, as described in Olson *et al.* (2012), Lehermeier *et al.* (2015), and de los Campos *et al.* (2015). The underlying rationale of such multivariate models is that marker effects, and more importantly the effects of causal mutations underlying traits, are likely to differ between subpopulations. Thus, allowing for different, albeit correlated, marker effects for each subpopulation in a GS model could provide more accurate GEBVs than models that only consider marker effects across subpopulations. When applied to a multi-breed dairy cattle data set in Olson *et al.* (2012), this multivariate model slightly outperformed a standard GS model fitted across all breeds in the data set. Similarly, Lehermeier *et al.* (2015) showed that the performance of the multivariate GS model relative to the other tested models varied depending upon the degree of subpopulation structure in the three tested crop panels, but consistently performed well. Finally, de los Campos *et al.* (2015) considered a Bayesian multivariate GS model that included marker main effects and marker x subpopulation two-way interaction effects that respectively enabled across- and within-subpopulation marker effect estimates, and observed that this model on average performed better than simpler GS models. Given the relatively high prediction accuracies we obtained from the PCA-only models (Figure 2), it is reasonable to hypothesize that the effect sizes of the markers and causal mutations differ between subpopulations of the Msi and Msa diversity panels. Accordingly, we would expect these multivariate GS models to estimate differing marker effects across subpopulations more accurately than the models we investigated, and that on average we would expect to observe a modest increase in prediction accuracies.

The CDmean procedure had a tendency to sample heavily from the subpopulation clusters located at the extremes of the PC biplots made for both the Msi and Msa individuals (Figures S6 and S7). Relative to the maize data analyzed by Rincent *et al.* (2012) and the wheat and rice data analyzed by Isidro *et al.* (2015), the degree of subpopulation structure present in the Msi and Msa diversity panels was most consistent with the rice data from Isidro *et al.* (2015). Consequently, the relative location of Msi and Msa individuals selected by the CDmean procedure were at similar positions on the PC biplots as what was observed in those for these rice data (compare Figures S7-S8 with Figure 5d-f in Isidro *et al.* 2015 for a visual assessment). Across all of the studies we conducted, the only instance where the CDmean procedure selected training sets that decisively outperformed an equally-sized training set of 200 randomly-selected individuals was in the fivefold cross validation study conducted within each diversity panel (summarized in Figure 2). Coupled with the performance of the CDmean-based training sets in the simulation studies (where population structure was not accounted for when randomly selecting QTN underlying simulated traits), we infer that training sets obtained from the CDmean procedure should outperform an equally-sized training set for (i) traits where subpopulation structure contributes substantially to the genomic sources of their variability and (ii) validation sets where subpopulation structure is roughly similar to that of the corresponding training sets. However, it is also important to emphasize that we never observed an instance where use of the CDMean procedure resulted in prediction accuracies that exceeded those from using all individuals in a training set. Thus, we recommend against using the CDmean procedure in practice and instead suggest that all available individuals be used as a training set.

### Contribution of low marker density to low prediction accuracies

A key limitation of our study is that we used only 5,140 markers common to all three panels to evaluate the ability of the GS models to predict breeding values of the observed traits. Given the rapid LD decay rate that we observed in both the Msi and Msa diversity panels (where pairwise LD decayed to nominal levels at distances less than 3 kb, Figure S8A-C), we conclude that the markers we used for GS did not provide sufficient density to tag enough causal variants; consequently, the observed prediction accuracies might be lower than expected with a denser marker set. This conclusion is in part justified given that prediction accuracies tended to be higher when Clark *et al.* (2019b) performed GS on the same Msi diversity panel using 46,177 SNPs. Thus, one avenue for future research would be to redo this study with a larger number of genome-wide markers. Similar to previous studies that evaluated GS prediction accuracy as a function of the number of markers (Lorenzana and Bernardo 2009; Asoro *et al.* 2011; Arruda *et al.* 2015), our expectation is that prediction accuracy would tend to increase as the number of markers increase. In particular, we expect that initially substantial gains in prediction accuracy will be followed by smaller increases, culminating in a plateau (and potentially a slight decrease) as the markers begin to tag a sufficient number of causal variants underlying the studied trait.

### Comparison of experimental and simulation genomic selection results

Extremely low and often negative prediction accuracies were obtained for the observed traits in the 09F2 panel when either one or both of the Msi or Msa panels were used to train the GS models (Figure 3). Our analysis expands upon previous studies conducted in maize (Windhausen *et al.* 2012; Brauner *et al.* 2020) and cattle (Raymond *et al.* 2018) by showing that adding the GEBVs calculated from training separate GS models within diversity panels, each from one of two different species, can also yield low prediction accuracies. However, one limitation of our analysis of the observed traits was that only one interspecific cross was available for evaluation, and we were therefore unable to observe how our results might differ across multiple interspecific F_2_ populations.

The lack of availability of such multiple F_2_ populations motivated our evaluation of prediction accuracies across 50 simulated interspecific populations, each of which have one randomly selected Msi parent and one randomly selected diploid Msa parent. Although these prediction accuracies were from simulated traits, they nevertheless provided additional insight into the ability of diversity panels to predict breeding values in interspecific crosses. For instance, we observed similar ranges of prediction accuracies regardless of the amount of non-additive genetic signals underlying the simulated traits. We also observed slightly higher prediction accuracies when the QTNs were common between Msi and Msa. These results suggest that prediction accuracies might be robust to variations in genetic architectures within and between panels, especially if predictions are made based on the sum of GEBVs fitted separately within the two diversity panels.

We noticed several major differences between GS prediction accuracies in the observed and simulated traits. Most importantly, prediction accuracies were noticeably lower in the observed traits than the simulated traits. It is likely that this result arose because the marker set used lacked the density to sufficiently tag the causal mutations underlying the observed traits (discussed previously). Another important difference was that for the simulated traits, adding the GEBVs together from the GS models trained separately in the panels were consistently yielded the highest prediction accuracies; meanwhile for the observed traits, this strategy never yielded the highest prediction accuracies. One hypothesis explaining this discrepancy was that the size of the genetic effects, and possibly the genetic architectures, of the observed traits substantially differed between the panels. This hypothesis is supported by the prediction accuracies observed in the simulation settings where one of the diversity panels contained the large-effect QTNs, while the other contained only small-effect QTN (Figure 4).

Thus, the inferences we made for the six observed traits are limited because we were unable to assess prediction accuracy for these traits across multiple F_2_ families. To fully assess whether or not our results for the six observed traits are generalizable to interspecific M×g material, multiple F_2_ families are needed. This important shortcoming motivated us to simulate multiple F_2_ populations, where each population was derived from randomly selected Msi and diploid Msa parents. Although not as useful as actual F_2_ populations, the simulation study provided additional preliminary insight into the paradigm of breeding interspecific M×g breeding material. As shown in Figure 4, we obtained the highest prediction accuracies in the simulated F_2_ populations whenever we added the GEBVs from the model trained in the Msi panel to those trained in the Msa panel. This suggests that a potential breeding strategy could be to produce interspecific F_2_ populations using Msi and Msa parents that are selected from GS models trained within their respective species. Preliminary simulation results suggest that such a strategy could yield prediction accuracies that are equivalent (after controlling for sample size differences between the F_2_ populations and the diversity panels) to those from GS models trained in multiple interspecific F_2_ populations, each with different parents (Figure S9). However, these preliminary results from simulated data need to be confirmed using real trait data on actual interspecific F_2_ populations. Such a follow-up study on real data will help determine if the gains in prediction accuracy from using these F_2_ populations are substantial enough to justify the additional time and resources needed to develop these populations.

### Implications of this work within the context of breeding M×g

The actual and simulated M×g F_2_ germplasm that we analyzed consisted of diploid individuals, and thus our inferences on the prospects of GS in M×g are most directly applicable to diploid F_2_ M×g material. Because Msa exists in diploid and tetraploid forms, there can be diploid, triploid, or tetraploid M×g, all of which have exhibited strong hybrid vigor (Clark *et al.* 2019a). As such, M×g F_2_ populations can help determine which regions of the genome are acting in an overdominant fashion for this heterosis (which may just be due to the linkage of dominant alleles) and which might be more advantageous to introgress directly from Msi or Msa. This can feed back into the generation of triploid and tetraploid Mxg if we were to create more complex hybrids, for example hybrids derived from fertile hybrid or introgressed individuals (*e.g.*, 2x M×g × 4x M×g = 3x M×g). Therefore, one logical extension of the work presented here would to use and modify approaches from other studies (*e.g.*, Bernal-Vasquez *et al.* 2017; Guo *et al.* 2019; Technow 2019; Beyene *et al.* 2019) to assess the ability of Msi, Msa, and/or non-diploid M×g populations to predict general and specific combining ability in Mxg hybrids.

In any case, GS is expected to benefit breeding in *Miscanthus* by facilitating early selection. Moreover, M×g often has greater yield-potential than either of its parental species (Clark *et al.* 2019a); thus, such hybrids are typically the focus of biomass cultivar development programs (Clifton-Brown *et al.* 2019). Depending on the target environment and how one balances the goal of preventing invasiveness via sterility *vs.* the goal of minimizing the cost of establishing production fields via direct seeding, the breeder’s objective may be to develop diploid, triploid or tetraploid M×g cultivars (Clifton-Brown *et al.* 2019). Population improvement within the parental species of M×g (*i.e.*, Msi and Msa) is anticipated to be an effective use of GS for developing germplasm pools that produce exceptional parental genotypes for cultivar development. Moreover, population improvement via GS within diploid and tetraploid F_2_ or backcross M×g populations will provide a full range of interspecific genomic compositions to test and from which to develop cultivars that are diploid, triploid or tetraploid. Thus, the work presented in this paper should serve as only the beginning of more complex future studies that evaluate the effective use of Msi, Msa, and M×g germplasm that is currently being developed for training the most efficient GS models for rapid genetic gain in M×g. Indeed, our findings suggest that more research is needed into addressing the question of optimal training set composition.

## Conclusion and recommendations

We ultimately conclude that using Msi and Msa diversity panels to train GS models for prediction of breeding values in an interspecific diploid F_2_ population is not an ideal strategy for increasing the genetic diversity of M×g. One potential reason for the low prediction accuracies we observed in the 09F2 population is the inherent difficulty in obtaining an ideal training set for an interspecific F_2_ cross. When analyzing the performance of GS in multiple maize breeding populations, Windhausen *et al.* (2012) showed that GS prediction accuracy for a targeted breeding population increased whenever the training sets were augmented with individuals from the same breeding population. This finding underscores the importance of including individuals in training sets that have approximately the same QTN in as those in the validation set. Indeed, we observed in our study that prediction accuracies were slightly higher for the simulated traits where the same QTN were segregating in both Msi and Msa, and hence these QTN were also segregating in the interspecific F_2_ populations (Figure 4). We therefore recommend that the best-suited training set for efficient GS of interspecific M×g populations should contain QTNs that are common in both the training and validation sets, and importantly are containing QTNs that are segregating between Msi and Msa. Because it is unlikely that QTNs are genotyped, we suggest taking efforts to ensure that individuals in the training and validation sets are as closely related as possible. Such a strategy will increase the genetic similarity between the training and validation sets, and hence increase the likelihood that that the trained GS model is accounting for QTNs that are segregating in the both of these sets.
